# *Ant1* mutant mice bridge the mitochondrial and serotonergic dysfunctions in bipolar disorder

**DOI:** 10.1038/s41380-018-0074-9

**Published:** 2018-06-11

**Authors:** Tomoaki M. Kato, Mie Kubota-Sakashita, Noriko Fujimori-Tonou, Fumihito Saitow, Satoshi Fuke, Akira Masuda, Shigeyoshi Itohara, Hidenori Suzuki, Tadafumi Kato

**Affiliations:** 1 Laboratory for Molecular Dynamics of Mental Disorders, RIKEN Center for Brain Science, Wako, Saitama, Japan; 2 Department of Pharmacology, Nippon Medical School, Tokyo, Japan; 3 Laboratory for Behavioral Genetics, RIKEN Center for Brain Science, Wako, Saitama, Japan; 4 Present Address: Department of Fundamental Cell Technology, Center for iPS Cell Research and Application, Kyoto University, Kyoto, Japan

**Keywords:** Neuroscience, Bipolar disorder, Genetics, Physiology

## Abstract

Although mitochondrial and serotonergic dysfunctions have been implicated in the etiology of bipolar disorder (BD), the relationship between these unrelated pathways has not been elucidated. A family of BD and chronic progressive external ophthalmoplegia (CPEO) caused by a mutation of the mitochondrial adenine nucleotide translocator 1 (*ANT1*, *SLC25A4*) implicated that *ANT1* mutations confer a risk of BD. Here, we sequenced *ANT1* in 324 probands of NIMH bipolar disorder pedigrees and identified two BD patients carrying heterozygous loss-of-function mutations. Behavioral analysis of brain specific *Ant1* heterozygous conditional knockout (cKO) mice using lntelliCage showed a selective diminution in delay discounting. Delay discounting is the choice of smaller but immediate reward than larger but delayed reward and an index of impulsivity. Diminution of delay discounting suggests an increase in serotonergic activity. This finding was replicated by a 5-choice serial reaction time test. An anatomical screen showed accumulation of COX (cytochrome c oxidase) negative cells in dorsal raphe. Dorsal raphe neurons in the heterozygous cKO showed hyperexcitability, along with enhanced serotonin turnover in the nucleus accumbens and upregulation of *Maob* in dorsal raphe. These findings altogether suggest that mitochondrial dysfunction as the genetic risk of BD may cause vulnerability to BD by altering serotonergic neurotransmission.

## Introduction

Bipolar disorder is a major mental disorder characterized by mania and depression. Dysregulation in both monoaminergic systems [1] and mitochondrial calcium signaling [2] have been proposed in the etiology of bipolar disorder. However, the relationship between these apparently unrelated metabolic signaling systems has not been elucidated [3]. Clinical studies showed that around 20% of patients with mitochondrial disease have comorbid bipolar disorder [4–6], whereas 0.38% of patients with bipolar disorder had mutations of *POLG* (polymerase γ) causative for mitochondrial disease [7]. In a family of bipolar disorder and autosomal dominantly inherited chronic external ophthalmoplegia (CPEO), the L98P mutation of *ANT1* (adenine nucleotide translocator 1, *SLC25A4*) was identified [8, 9]. These findings suggest that central nervous system involvement in mitochondrial disease caused by *ANT1* mutations confers a risk of bipolar disorder. *ANT1* is named for its function as a translocator of adenosine triphosphate and adenosine diphosphate across the mitochondrial inner membrane. However, its modulatory role in the mitochondrial permeability transition pore (mPTP) has also drawn attention [10–12]. The mPTP plays a role in regulated cell death, and transient opening of the mPTP also regulates mitochondrial calcium signaling [13], which is consistent with the well-known calcium dysregulation hypothesis of bipolar disorder [14].

In this study, we searched for *ANT1* mutations in patients with bipolar disorder, and identified two independent loss of function (LOF) mutations of *ANT1*. We investigated the relationship between heterozygous loss of function of *ANT1* and bipolar disorder by generating a brain specific *Ant1* conditional knockout (cKO) mouse. By behavioral screening, we identified that the heterozygous mice showed diminished delay discounting, that is the choice of smaller but immediate reward than larger but delayed reward and an index of impulsivity [15]. Consistent with this finding, the mice had enhanced serotoninergic activity. These findings together shed new light on the mechanism of how *ANT1* mutations may confer a risk for BD.

## Materials and methods

The study was approved by the Wako first ethics committee of RIKEN. All animal care and experimental procedure were in accordance with the guidelines for proper conduct of animal experiments published by Science Council of Japan and approved by RIKEN Wako Animal Experiment Committee. Methods and materials are described in detail in Supplemental Experimental Procedures.

### Subjects

All four exons of the *SLC25A4* gene were sequenced by PCR-direct sequencing in 324 probands of NIMH Genetics Initiative bipolar disorder pedigrees. Their diagnosis was bipolar I (*n* = 304), bipolar II disorder (*n* = 17), or schizoaffective disorder, bipolar type (*n* = 3).

### Animals

Floxed exon 2–3 of *Slc25a4* mouse line (*Slc25a4*^tm1a(EUCOMM)Wtsi^) was obtained from International Knockout Mice Consortium (IKMC). Flp-transgenic mouse line (B6 ^Tg(cat-Flpe)36^) was previously generated [16]. Nestin-Cre transgenic mouse line (B6.Cg^Tg(Nes-cre)1Kln/J^) was obtained from Jackson laboratory. Using these mice lines, heterozygous and homozygous cKO mice of *Slc25a4* (*Slc25a4*^*fl/fl*^ or *Slc25a4*^*fl/+*^) (*Ant1* cKO mice) were generated.

### Staining

Staining methods and antibodies are described in detail in Supplementary Methods.

Fluorescent in situ hybridization was conducted as previously described [17]. Images were captured by a confocal microscope IX81 with FV1000 (Olympus Corporation, Tokyo, Japan), Observer Z1 with AxioVision 4.6 (Zeiss, Oberkochen, Germaney) or Nano Zoomer Digital Pathology system (Nanozoomer 2.0RS, Hamamatsu Photonics, Hamamatsu, Japan).

### Mouse behavioral screening with IntelliCage

The IntelliCage apparatuses (NewBehavior AG, Zurich, Switzerland) were used for behavioral screening as described previously [18, 19]. Male mice including 8 heterozygous cKO mice (*Slc25a4*^*fl/+*^; Nes-Cre+), 10 homozygous cKO mice (*Slc25a4*^*fl/fl*^; Nes-Cre+) and 6 controls (*Slc25a4*^*fl/+*^ or *Slc25a4*^*fl/fl*^; Nes-Cre−), which were 20–27 week old, were used.

### 5-choice serial reaction time task (5-CSRTT)

The 5-CSRTT operant chamber (O’HARA & Co., Tokyo, Japan) was used as previously described with minor modification [20]. Male mice including 8 controls (*Slc25a4*^*fl/+*^ or *Slc25a4*^*fl/fl*^; Nes-Cre−), 8 heterozygous cKO mice (*Slc25a4*^*fl/+*^; Nes-Cre+) and 8 homozygous cKO mice (*Slc25a4*^*fl/fl*^; Nes-Cre+), which were 8–13 week old at the beginning of training, were used for the analysis.

### Quantification of mtDNA deletion and mtDNA copy number

Partially deleted mitochondrial DNA (ΔmtDNA) and copy number of mtDNA was measured by quantitative PCR methods using SYBR Premix Ex Taq Kit (Takara Bio, Otsu, Japan) and QuantStudio 12 K Flex (Thermo Fisher Scientific, Waltham, MA) as described [21]. For quantification of mtDNA deletion 30–39 week old male mice were used. For mtDNA copy number analysis, 54–56 week old male mice were used. Control mice were *Slc25a4*^fl/+^ or *Slc25a4*^fl/fl^ without Nestin-Cre.

### Quantification of *Ant1/Ant2* mRNAs

mRNA expression of *Ant1* (*Slc25a4*) and *Ant2* (*Slc25a5*) were measured by quantitative PCR methods using SYBR Premix Ex Taq Kit (Takara Bio, Kusatsu, Japan) and QuantStudio 12 K Flex (Thermo Fisher Scientific, Waltham, MA). For these analysis, female 78–114 week old mice were used (*n* = 3 for each group).

### Measurement of calcium retention capacity

Brain mitochondria were isolated using a discontinuous Percoll gradient developed by Sims [22] with minor modifications [23]. Extra-mitochondrial free Ca^2+^ concentration ([Ca^2+^]_exm_) was monitored with 200 nM Calcium Green-5N (Thermo Fisher Scientific, Waltham, MA) (Ex 480 nm, Em 540 nm) in a 96 well plate at 30 °C in a Drug Screening System (FDSS 3000, Hamamatsu Photonics, Hamamatsu, Japan). To evaluate the CRC, 10 µl of Ca^2+^ solution was repeatedly added at 1-min intervals. Heterozygous cKO mice (*Slc25a4*^*fl/+*^; Nes-Cre+) (*n* = 4), homozygous cKO mice (*Slc25a4*^*fl/fl*^; Nes-Cre+) (*n* = 4), and control mice (*Slc25a4*^*fl/fl*^; Nes-Cre−) (*n* = 3) aged 8–27 weeks were used for this analysis.

### Quantification of monoamine in tissue by HPLC

Dopamine, noradrenaline and serotonin and their metabolites were measured by HPLC with an EICOMPAK SC-5ODS with electrochemical detector ECD-300 (Eicom Corporation, Kyoto, Japan). In this experiment, control mice included *Slc25a4*^+/+^; Nestin-Cre+ and *Slc25a4*^+/+^, *Slc25a4*^fl/+^ or *Slc25a4*^fl/fl^ without Nestin-Cre. For this experiment, 88–103 week old male mice were used.

### Electrophysiological analysis

Brain slices for experiments were prepared from 10–12-week-old, male mice as described previously [24]. Whole cell patch-clamp recordings were acquired and controlled using the Axon 700B Multiclamp amplifer (Molecular Devices, CA, US) and pClamp11 acquisition software (Molecular Devices, CA, US). For this analysis, heterozygous *Ant1* cKO mice (*Slc25a4*^*fl/+*^; Nes-Cre+) (*n* = 4) and control mice (*Slc25a4*^*fl/+*^ without Nes-Cre) (*n* = 3) aged 8–12 week old were used.

### Statistical analysis

Data were analyzed by Prism 4 (Graphpad softoware Inc., San Diego, CA), IBM SPSS Statistics 20 (IBM Japan, Tokyo, Japan), “R” (https://www.r-project.org/) or Kyplot (Kyence, Tokyo, Japan). For genetic association analysis, Fisher’s exact probability test was used. In the comparison between the heterozygous or homozygous cKO mice and control mice, Student *t*-test was used. For place learning task and delay discounting task in IntelliCage, repeated measures ANOVA with Bonferroni’s post hoc test was used with main effects of genotype and delay or day.

## Results

### Identification of loss of function mutations in bipolar patients

We sequenced the *ANT1* gene in 324 NIMH probands with bipolar disorder and identified two patients carrying LOF mutations. One patient had a stop codon mutation p.Q85X, a substitution of C to T at Chr 4: 185,144,905 [hg38] while the other had p.Q175RfsX38, a single nucleotide deletion at Chr 4: 185,145,174, causing a frameshift and premature stop codon (Supplementary Figures 1A-B), which was reported in a pedigree of recessive cardiomyopathy with comorbid depression and anxiety [25]. The frequency of LOF mutations (gain of stop codon or frame shift) for *ANT1* in BD (2/324, 0.61%) was significantly higher than that in the exome and genome data in the gnomAD database (10 of 128,632 [2 stop codons and 8 frameshift mutations], 0.000069%, Fisher’s exact probability test, *P* *=* 0.00040, odds ratio = 79.7 [95%CI: 8.4–374.4]). Both mutations were on exon 3 and were thus predicted to undergo nonsense-mediated mRNA decay. Even when cDNAs of the predicted mutant mRNA encoding truncated proteins were constructed, protein expression of these mutants was markedly reduced in Neuro 2A cells (Supplementary Figure 1C). Although the two mutations did not show complete cosegregation with BD in the two pedigrees partly because of bilinear transmission (Supplementary Figure 1D), the significant association with high odds ratio suggested the role of these LOF mutations as a genetic risk factor for BD.

### Generation of brain-specific *Ant1* cKO mice

**Fig. 1:**
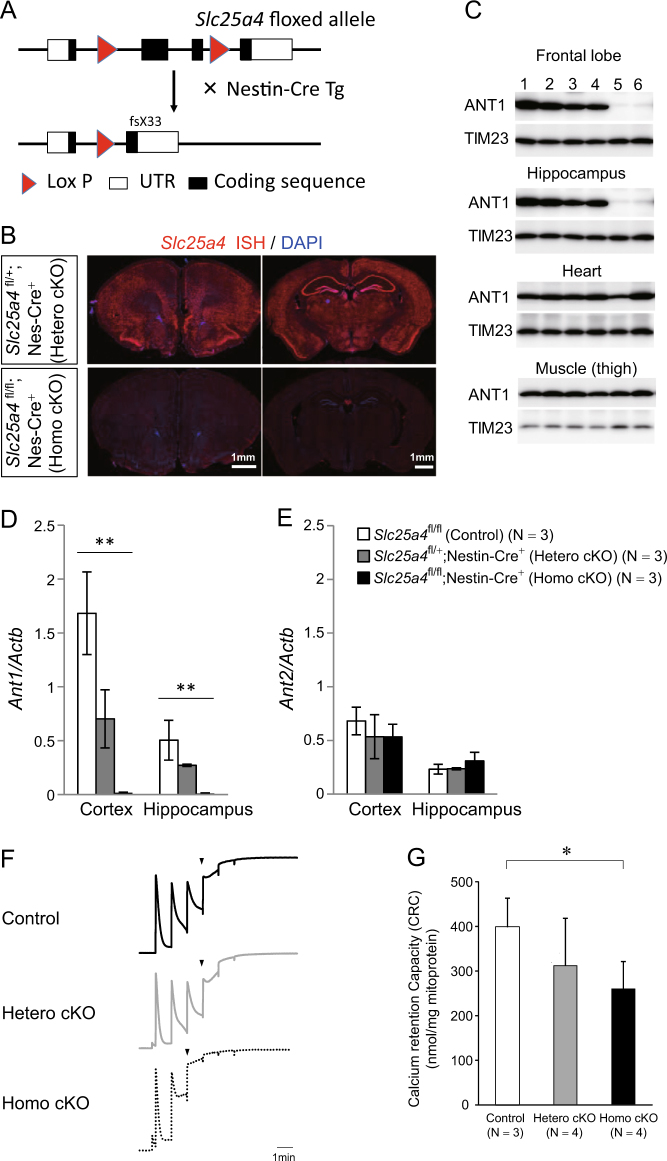
Generation of brain-specific *Ant1* conditional knockout (cKO) mice. **a** Structures of floxed *Ant1* (*Slc25a4*) allele and conditionally knocked out (cKO) allele of *Ant1*. **b** In situ hybridization for *Ant1* (red) and DAPI (blue) staining in the brains of heterozygous and homozygous *Ant1* cKO mice. No mRNA expression was detected in homozygous cKO mice. **c** Western blotting of ANT1 and TIM23 (internal control for inner mitochondrial membrane) of brain tissues and muscle in heart or thigh from male (1, 3, 5) and female (2, 4, 6) control mice (1, 2) and heterozygous (3, 4) and homozygous (5, 6) *Ant1* cKO mice. **d, e** Quantitative PCR analysis for *Ant1* (**d**) and *Ant2* (*Slc25a5*) (**e**) in the brains of control mice and heterozygous and homozygous *Ant1* cKO mice. **P *<* 0.005 by one way ANOVA. Post hoc test indicates significant difference of *ANT1* between all three groups for cortex and a significant difference between homozygous cKO and controls for hippocampus. No significant group difference was found for *ANT2*. **f** Representative charts of the experiments to measure calcium retention capacity (CRC). Vertical axis indicates Calcium green fluorescence intensity that reflects Ca^2+^ concentration of extra-mitochondrial fluid. Decay phase of each rise of extra-mitochondrial Ca^2+^ concentration reflects the uptake of Ca^2+^ by mitochondria. Arrow heads indicate the timing of opening of mitochondrial permeability transition pore (mPTP). Mitochondria from homozygous *Ant1* cKO mice showed earlier mPTP opening, indicating smaller CRC. **g** Effect of loss of *Ant1* on the mPTP opening. The vertical axis indicates the CRC. The data represents mean ± SEM. *P *<* 0.05

*ANT1* mutations are known to cause a neuromuscular disorder, CPEO, and thus behavioral analyses must be performed using brain-specific mutant mice. We crossed floxed *Ant1* mice (*Slc25a4*^fl/fl^ or *Slc25a4*^fl/+^) with Nestin-Cre transgenic mice to generate a brain-specific cKO *Ant1* mouse (Fig. 1a). We verified that the homozygous *Ant1* cKO mice (*Slc25a4*^fl/fl^; Nestin-Cre+) have no *Ant1* mRNA (Fig. 1b) and protein (Fig. 1c) expression in the brain, and heterozygous *Ant1* cKO mice (*Slc25a4*^fl/+^; Nestin-Cre+) have reduced *Ant1* mRNA (Fig. 1d) compared to control mice (*Slc25a4*^+/+^; Nestin-Cre+, *Slc25a4*^fl/+^ or *Slc25a4*^fl/fl^; without Nestin-Cre). No compensatory upregulation of *Ant2* mRNA was observed (Fig. 1e).

To examine the functional consequences of the loss of *ANT1*, we isolated mitochondria from *Ant1* cKO mice. The mitochondrial calcium retention capacity was significantly lower in homozygous *Ant1* cKO mice (*P* *=* 0.02) (Figs. 1f, g) suggesting that the mitochondria of homozygous *Ant1* cKO mice are vulnerable to the mPTP opening. The calcium retention capacity of heterozygous *Ant1* cKO mice did not significantly differ from controls.

Because the identified patients with bipolar disorder were heterozygous for LOF mutations in *ANT1*, we performed the murine behavioral characterization in heterozygous *Ant1* cKO mice to model the human disorder. Homozygous cKO mice were examined as a reference control, and *Slc25a4*^fl/+^ or *Slc25a4*^fl/fl^ without Nestin-Cre, or *Slc25a4*^+/+^; Nestin-Cre+ were also examined as controls.

### Behavioral phenotypes of brain-specific *Ant1* cKO mice

**Fig. 2:**
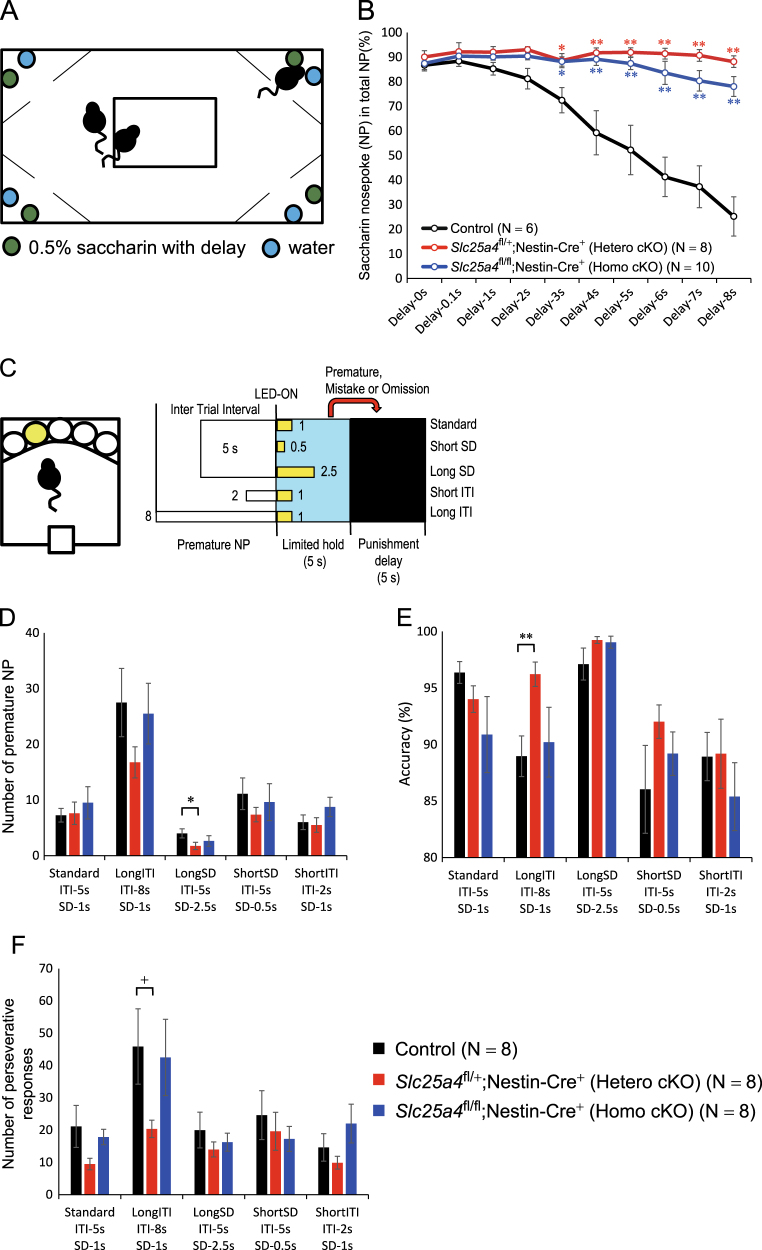
Behavioral analysis of *Ant1* cKO mice by IntelliCage and five-choice serial reaction time test (5-CSRTT). **a** Schematic drawing of delay discounting task in IntelliCage. The actions such as nose poking and licking of mice in each corner to drink running water (cyan) or 0.5% saccharin containing water (green) can be automatically recorded. **b** Percentages of nose poke counts for saccharin water in total nose poke counts in each day without or with activation delay after corner entry. Nose poke ratios for saccharin water of heterozygous (*n* = 8, red) and homozygous (*n* = 10, blue) conditional knockout mice were higher than control mice (*n* = 6, black) in long delay (≥3 s). Each circle represents mean and error bars indicate standard error of mean (SEM). **P *<* 0.005; *P *<* 0.05 (Bonferroni’s post hoc test after two-way repeated measures ANOVA). Red and blue asterisks indicate the comparisons of control vs heterozygous cKO, or control vs homozygous cKO, respectively. **c** Schematic drawing of 5-CSRTT. A sugar pellet was delivered to food magazine by a correct nose poke at an illuminated hole among five holes during limited hold. LED which was a sign for a correct hole was turned on after inter trial interval (ITI) for the defined period in each test. Premature or mistake nose poke and omission trial will lead to additional punishment delay in which room light was shut down for 5 s. **d** Number of premature nose poke in heterozygous mutant mice was significantly decreased compared with that in control mice in long stimulus duration test. **e** Accuracy (percentage of correct nose poke / (correct nose poke + mistake)) was significantly higher in heterozygous *Ant1* cKO mice compared with controls in long-ITI test. **f** Number of preservative responses was lower in heterozygous *Ant1* cKO mice compared with controls in long-ITI test. **P *<* 0.005; *P *<* 0.05, ^+^*P* *=* 0.05. *SD* stimulus duration, *ITI* inter-trial interval, *NP* nose poke

We performed behavioral screening using the IntelliCage (Fig. 2a). Indices for spatial learning, reverse learning, and attention did not differ between genotypes (Supplementary Figures 2A-E), but the heterozygous cKO mice showed a significantly decreased delay discounting (Fig. 2b). The preference for saccharin did not differ between genotypes (*F* = 1.0, *P* = 0.36 by one way ANOVA) (Supplementary Figure 2F), suggesting that this finding is not due to altered reward value. Homozygous *Ant1* cKO mice also showed a similar behavioral phenotype (Fig. 2b), although more similar to controls than heterozygous cKO mice.

To further characterize the behavioral phenotypes of heterozygous *Ant1* cKO mice, we performed a 5-choice serial reaction time test (5-CSRTT), an established test to measure impulsivity [26] as a reflection of enhanced delay discounting (Figs. 2c–f, Supplementary Figures 2G-I). In this test, the number of premature nose pokes during long stimulus duration trials, which is an index of impulsivity, was significantly lower in heterozygous cKO mice than control mice (Fig. 2d). A decrease of impulsivity is equivalent to a decrease in delay discounting shown by the IntelliCage (Fig. 2b). The heterozygous cKO mice also showed better accuracy (Fig. 2e) and a lower number of perseverative responses than control mice in the long inter-trial interval trials (Fig. 2f). Homozygous cKO mice did not show similar phenotypes in the 5-CSRTT for unknown reasons.

### Anatomical screen

**Fig. 3:**
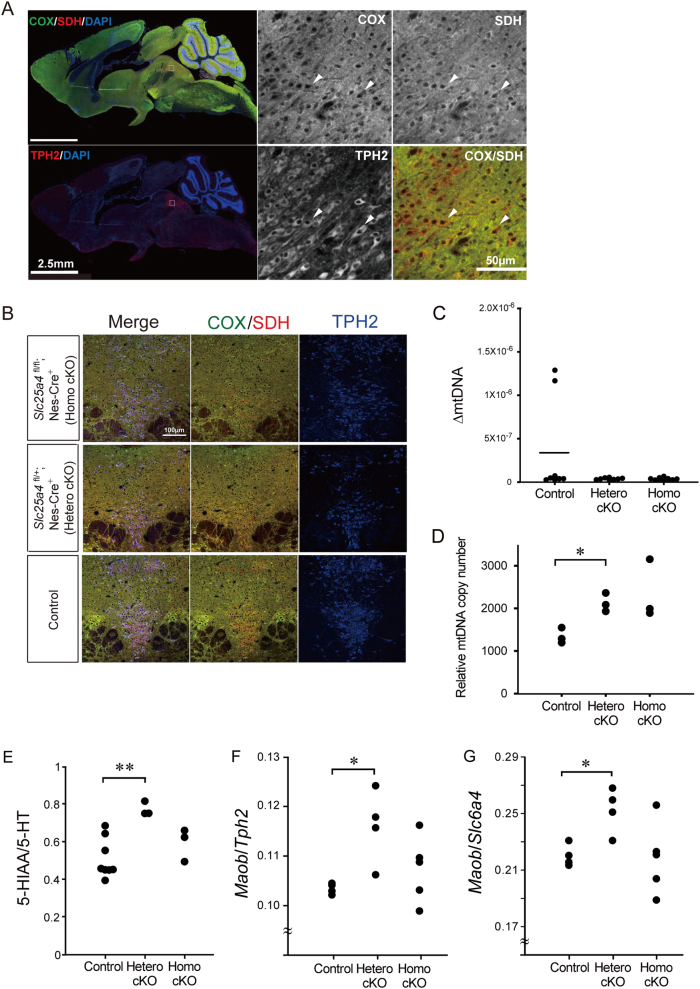
Anatomical screen by COX/SDH immunostaining of the brain suggested the role of dorsal raphe serotonergic neurons. **a** COX/SDH staining of a sagittal section of a 34 week old male homozygous *Ant1* cKO mouse. COX negative cells are seen as cells with red cytosol (positive signal of SDH [red] and no signal of COX [green]). Images were obtained by NDP and Axiovision. **b** COX/SDH staining of homozygous *Ant1* cKO mouse (upper), heterozygous *Ant1* cKO mouse (middle) and control mouse (bottom) (13 week old). Triple staining images for COX (green), SDH (red) and TPH2 (blue) indicate that COX negative cells are present in TPH2 positive serotonergic neurons. Images were obtained by confocal microscopy (FV1000). **c** Quantification of mtDNA deletions (ΔmtDNAs) by quantitative PCR. The bar indicates average. There was no significant difference of ΔmtDNAs between heterozygous *Ant1* cKO mice (*n* = 8) and control mice (*n* = 8). The homozygous cKO mice (*n* = 10) also did not accumulate mtDNA deletions. **d** Quantification of mtDNA copy number. A nuclear gene RNaseP was used as a reference. Heterozygous *Ant1* cKO mice (*n* = 3) had significantly higher levels of mtDNA than control mice (*n* = 3) (*P* *<* 0.01). The homozygous cKO mice (*n* = 3) also showed a tendency of elevation of mtDNA levels (*P* *=* 0.07). **e** The 5HIAA (5-hydroxyindole acetic acid)/5-HT (5-hydroxytryptamine, serotonin) ratio indicating turnover of serotonin is accelerated in heterozygous *Ant1* cKO mice compared with control mice. **P *<* 0.005. Homozygous *Ant1* cKO did not significantly differ from controls (*P* *=* 0.42). **f**, **g** *Maob* mRNA levels in dorsal raphe of *Ant1* KO mice. Heterozygous *Ant1* cKO mice showed significantly increased *Maob*/*Tph2* (**f**) and *Maob*/*Slc6a4* (**g**) ratios compared with control mice

We searched for brain regions with mitochondrial dysfunction due to the heterozygous knockout of *Ant1* by performing COX (cytochrome c oxidase)/SDH (succinate dehydrogenase) co-staining. COX is a mtDNA-encoded protein whereas SDH is a nuclear-encoded mitochondrial protein, and COX-negative cells were detected in a model mouse of mitochondrial disease [27]. In a sagittal section of aged homozygous *Ant1* cKO mice, COX negative cells were detected preferentially in the dorsal raphe (DR) (Fig. 3a) and heterozygous *Ant1* cKO mice also showed COX negative cells in the DR (Fig. 3b). Unexpectedly, however, a similar accumulation of COX negative cells was also detected in the DR of wild type mice (Fig. 3b). In this region, mtDNA deletions (ΔmtDNA) were not detectable both in cKO and control mice (Fig. 3c). Thus, the DR may have a selective vulnerability to mitochondrial dysfunction unrelated to the accumulation of ΔmtDNA, and the phenotype of the mutant mice might be caused by an interaction of the genotype and a general vulnerability of DR neurons to mitochondrial dysfunction. The copy number of mtDNA was significantly increased in DR of heterozygous cKO mice compared with control mice (Fig. 3d).

### Serotonergic dysfunction in *Ant1* KO mice

The activation of serotonergic neurons reportedly attenuates delay discounting [28], and therefore we examined serotonin turnover in the nucleus accumbens, which is innervated by DR serotonergic neurons and regulates impulsivity [29]. We found that serotonin turnover was enhanced in the nucleus accumbens of heterozygous KO mice (Fig. 3e). There was no significant difference of the serotonin turnover between Nestin-Cre and wild type mice (0.55 ± 0.07 vs 0.75 ± 0.17, respectively, *P* *=* 0.15, *n* = 3 for both groups). We also analyzed the gene expression level of *Maob* (monoamine oxidase B) that encodes a monoamine metabolizing enzyme on the mitochondrial outer membrane enriched in the DR [30]. To normalize expression levels of *Maob* by the number of serotonergic neurons within the sample, a serotonin neuron specific gene, *Tph2* or *Slc6a4*, was used for a reference. We found that *Maob* mRNA was significantly increased in the DR of heterozygous *Ant1* cKO mice (*P* *<* 0.05) (Figs. 3f, g) consistent with the elevated turnover of serotonin in the nucleus accumbens.

**Fig. 4:**
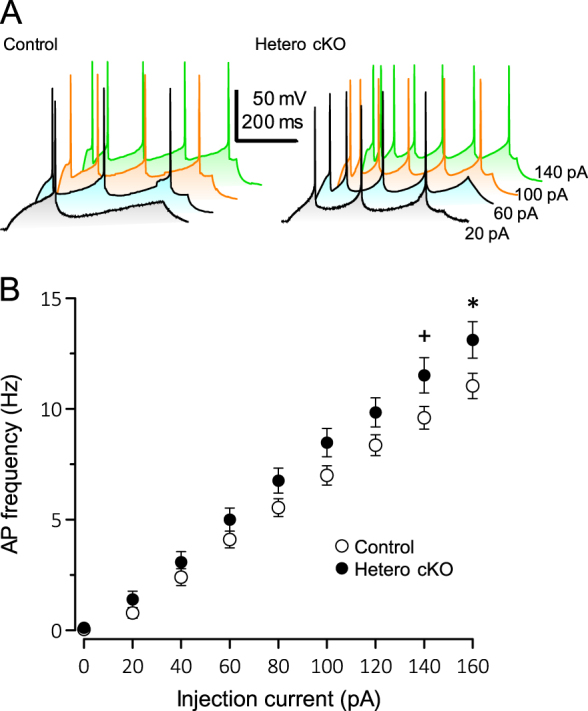
DR serotonergic neurons in heterozygous *Ant1* cKO mice show increased excitability. **a** Representative action potentials (APs) of DR neurons of the control and heterozygous *Ant1* cKO mice that were generated by injecting currents of 20 pA (black), 60 pA (blue), 100 pA (orange), and 140 pA (green), respectively. **b** Pooled data of input-output relationship curves of DR serotonergic neurons in the control (open circles, *n* = 43) and *Ant1* cKO (filled circles, *n* = 44). APs were generated by injecting current steps from 0–160 pA in increments of 20 pA for 500 ms. The frequency of AP generation were plotted. ^+^*P* *=* 0.05; *P *<* 0.05 by Student *t*-test

To test whether serotonergic neurons were activated in the *Ant1* cKO mice, we performed electrophysiological recordings from midbrain slices containing the DR. As shown in Supplementary Table 1, both basic membrane properties and action potential properties of serotonergic neurons at the midline of the DR did not show significant differences between genotypes. However, the input-output relationship curves, which show the generation of action potentials by current injection was steeper in heterozygous *Ant1* cKO mice than that of controls (Fig. 4) indicating that serotonergic neurons of heterozygous *Ant1* cKO mice are more excitable.

## Discussion

In this study, we identified two patients with bipolar disorder carrying loss of function mutations in *ANT1* (Supplementary Figure 1). Brain-specific heterozygous cKO mice of *Ant1* showed diminished delay discounting or reduced impulsivity by two behavioral tests (Fig. 2). Enhanced serotonin turnover (Fig. 3e) and hyperactivity of serotonergic neurons (Fig. 4) were consistent with the behavioral phenotype [28]. Increased mtDNA copy number in DR (Fig. 3d) might reflect an increased energy demand in the DR, and upregulation of *Maob* mRNA is considered a compensatory upregulation associated with enhanced serotonergic activity. These findings, together, suggest that the heterozygous loss of *Ant1* in the brain causes a hyperserotonergic state and associated behavioral phenotypes.

The mechanism of how the heterozygous loss of function of *Ant1* causes serotonergic hyperactivity is not known. Elevated intracellular calcium associated with depolarization is sequestered by mitochondria. The accumulation of intramitochondrial calcium results in a transient opening of the mPTP [31]. Ant1 reportedly has a modulatory effect on the mitochondrial permeability transition pore (mPTP) [10]. An altered mPTP function associated with heterozygous loss of *Ant1* might affect the excitability of DR neurons.

A previous study reported exaggerated corticosterone responses to stress in homozygous conventional *Ant1* KO mice [32]. Enhanced serotonergic function might also underlie this finding, because serotoninergic stimulation is known to activate the hypothalamic-pituitary-adrenal axis [33].

Why are serotonergic neurons preferentially affected by the heterozygous loss of *Ant1*? Monoamines are metabolized by monoamine oxidases (MAOs) on the mitochondrial outer membrane. Metabolism of monoamines by MAOs is accompanied by the generation of hydrogen peroxide, a reactive oxygen species [30]. Enhanced serotonin release by 3,4-methylenedioxymethamphetamine (MDMA) can decrease COX protein levels, which can be rescued by an MAO-B inhibitor [34]. We speculate that serotonergic neurons may be intrinsically vulnerable to mitochondrial dysfunction, and the hyperserotonergic state may further impair mitochondrial function in a vicious cycle. The results of the COX/SDH staining in this study support this hypothesis. The degeneration of DR serotonergic neurons is frequently seen in Parkinson’s disease, in which mitochondrial dysfunction is implicated, and this might at least partly explain the non-motor symptoms of this disease [35]. In Parkinson’s disease, 8-hydroxyguanosine is accumulated in substantia nigra dopaminergic neurons, and this is also true for the DR [36]. Thus, the mitochondrial dysfunction phenotype due to the heterozygous cKO of *Ant1* is predominantly seen in serotonergic neurons, which may have an intrinsic vulnerability to mitochondrial dysfunction. The mechanism of how COX immunoreactivity is reduced in the DR is not known because the present study did not show an increase of ΔmtDNA levels or a decrease of mtDNA copy number. Reduction of COX immunoreactivity in DR neurons might be regulated by other types of mtDNA abnormalities and/or at the protein level [37].

Mitochondrial dysfunction has been implicated in bipolar disorder based on several lines of evidence [2] including altered energy metabolism detected by magnetic resonance spectroscopy [38], comorbidity with mitochondrial diseases [4, 7], and findings in postmortem brains including an accumulation of ΔmtDNAs [39, 40], altered gene expression of mitochondria-related genes [41], altered morphology of mitochondria [42], and decreased activity of mitochondrial complex I [43]. On the other hand, serotonergic dysfunction in bipolar disorder has been implicated by evidence including the mania-inducing effect of antidepressants that inhibits the serotonin transporter and thereby activates serotonin [44], the efficacy of atypical antipsychotics that inhibits serotonergic neurotransmission [45], altered mRNA expression levels of serotonergic receptors in postmortem brain [46], altered DNA methylation of serotonin transporter gene [47], altered serotonin transporter binding in the brain by positron emission tomography [48], and levels of cerebrospinal fluid metabolites, among others [14]. The present findings provide a potential missing link between these two lines of evidence. Because the two LOF mutations identified in this study were not cosegregated with bipolar disorder in the two pedigrees, they are not “causative” mutations. However, their significant association suggests that the heterozygous LOF mutations of *ANT1* confer the risk of bipolar disorder.

There are several limitations in this study. Notably, it is unknown why homozygous *Ant1* cKO mice do not show behavioral alterations in some experiments. However, such non-linear dynamics are inherent to complex biological systems such as the brain. Secondly, Nestin-Cre mice reportedly have some behavioral alterations [49], which could in principle confound the results. However, we verified that this transgene did not affect serotonin turnover and excitability of DR serotonergic neurons (Supplementary Figure 3). Thirdly, behavioral analysis is confounded by genetic background [50]. Although all the mice used were generated and/or kept under the background of C57BL/6, there are subtle behavioral differences even among C57BL/6 substrains [51]. We therefore used the mice after backcrossing with C57BL/6J, though the number of generations might not be enough to rule out a possible effect of substrains. Finally, the results in calcium retention capacity are not consistent with previous studies that showed a loss of *Ant1* causing an increase in calcium retention capacity [52]. This discrepancy might be due to differences in experimental conditions or mouse strains.

In summary, our current findings suggest that mitochondrial dysfunction caused by heterozygous loss of *Ant1* can cause alterations of serotonergic activity, a first step toward understanding the complex neurobiological processes underlying bipolar disorder subtypes.

## Electronic supplementary material

Supplementary information
