# Supplementary material for: Ant1 mutant mice bridge the mitochondrial and serotonergic dysfunctions in bipolar disorder
Source: Mol Psychiatry. 2018 Jun 11;23(10):2039–49. doi: 10.1038/s41380-018-0074-9 (PMC6250678; doi:10.1038/s41380-018-0074-9)
Supplement: Supplementary file 1 — Supplementary information [file 41380_2018_74_MOESM1_ESM.pdf]

Supplementary information for

Ant1 mutant mice bridge the mitochondrial and serotonergic dysfunctions in bipolar disorder

Tomoaki M. Kato[1,4], Mie Kubota-Sakashita[1], Noriko Fujimori-Tonou[1], Fumihito Saitow[2], Satoshi Fuke[1], Akira Masuda[3], Shigeyoshi Itohara[3], Hidenori Suzuki[2], Tadafumi Kato[1]

[1] Laboratory for Molecular Dynamics of Mental Disorders, RIKEN Center for Brain Science, Wako, Saitama, Japan

[2] Department of Pharmacology, Nippon Medical School, Tokyo, Japan

[3] Laboratory for Behavioral Genetics, RIKEN Center for Brain Science Wako, Saitama, Japan

[4] Present Address: Department of Fundamental Cell Technology, Center for iPS Cell Research and Application, Kyoto University, Kyoto, Japan

The PDF file includes:

Supplementary Materials and Methods

Legends for Supplementary Figures 1-3

References for Supplementary Materials and Methods

Supplementary Figures 1-3

Supplementary Table 1-2

Supplementary Materials and Methods

Subjects

We analyzed 324 probands of NIMH Genetics Initiative bipolar disorder pedigrees including 194 females and 130 males. Their diagnosis was bipolar I (n=304), bipolar II disorder (n=17), or schizoaffective disorder, bipolar type (n=3). Mean age of probands with a record of age was 33.9 ± 9.8 (mean ± SD) years old.

The DNA samples were originally selected for trio analysis among NIMH pedigrees. The trios were selected based on the following criteria. 1) DNA is available for trio (a proband and the parents). 2) If more than one trios were found in one pedigree, bipolar I disorder was selected. If no, bipolar II or schizoaffective disorder, bipolar type were also selected. 3) If more than one trios of the same diagnosis were found in one pedigree, the trio was selected from a younger generation. 4) If more than one trios were available in one generation, an older sibling or a subject nominated as proband was selected.

Data and biomaterials were collected in four projects that participated in the National Institute of Mental Health (NIMH) Bipolar Disorder Genetics Initiative. From 1991 to 1998, the principal investigators and co-investigators were Indiana University, Indianapolis, IN, U01 MH46282, John Nurnberger, M.D., Ph.D., Marvin Miller, M.D., and Elizabeth Bowman, M.D.; Washington University, St. Louis, MO, U01 MH46280, Theodore Reich, M.D., Allison Goate, Ph.D., and John Rice, Ph.D.; Johns Hopkins University, Baltimore, MD, U01 MH46274, J. Raymond DePaulo Jr., M.D., Sylvia Simpson, M.D., M.P.H., and Colin Stine, Ph.D.; NIMH Intramural Research Program, Clinical Neurogenetics Branch, Bethesda, MD, Elliot Gershon, M.D., Diane Kazuba, B.A., and Elizabeth Maxwell, M.S.W.

Data and biomaterials were collected as part of 10 projects that participated in the NIMH Bipolar Disorder Genetics Initiative. From 1999 to 2003, the principal investigators and co-investigators were Indiana University, Indianapolis, R01 MH59545, John Nurnberger, M.D., Ph.D., Marvin J. Miller, M.D., Elizabeth S. Bowman, M.D., N. Leela Rau, M.D., P. Ryan Moe, M.D., Nalini Samavedy, M.D., Rif El-Mallakh, M.D. (at University of Louisville), Husseini Manji, M.D. (at Wayne State University), Debra A. Glitz, M.D. (at Wayne State University), Eric T. Meyer, M.S., Carrie Smiley, R.N., Tatiana Foroud, Ph.D., Leah Flury, M.S., Danielle M. Dick, Ph.D., Howard Edenberg, Ph.D.; Washington University, St. Louis, MO, R01 MH059534, John Rice, Ph.D., Theodore Reich, M.D., Allison Goate, Ph.D., Laura Bierut, M.D.; Johns Hopkins University, Baltimore, MD, R01 MH59533, Melvin McInnis, M.D., J. Raymond DePaulo Jr., M.D., Dean F. MacKinnon, M.D., Francis M. Mondimore, M.D., James B. Potash, M.D., Peter P. Zandi, Ph.D., Dimitrios Avramopoulos, and Jennifer Payne; University of Pennsylvania, Philadelphia, R01 MH59553, Wade Berrettini, M.D., Ph.D.; University of California at Irvine, R01 MH60068, William Byerley,

M.D., and Mark Vawter, M.D.; University of Iowa, Iowa City, R01 MH059548, William Coryell, M.D., and Raymond Crowe, M.D.; University of Chicago, IL, R01 MH59535, Elliot Gershon, M.D., Judith Badner, Ph.D., Francis McMahon, M.D., Chunyu Liu, Ph.D., Alan Sanders, M.D., Maria Caserta, Steven Dinwiddie, M.D., Tu Nguyen, Donna Harakal; University of California at San Diego, R01 MH59567, John Kelsoe, M.D., Rebecca McKinney, B.A.; Rush University, Chicago, IL, R01 MH059556, William Scheftner, M.D., Howard M. Kravitz, D.O., M.P.H., Diana Marta, B.S., Annette Vaughn-Brown, M.S.N., R.N., and Laurie Bederow, M.A.; NIMH Intramural Research Program, Bethesda, MD, 1Z01MH002810-01, Francis J. McMahon, M.D., Layla Kassem, Psy.D., Sevilla Detera-Wadleigh, Ph.D., Lisa Austin, Ph.D., Dennis L. Murphy, M.D.

In addition, families were contributed by Dr. Carlos Pato at the University of Southern California and his staff. This work was sponsored by NIMH grants MH52618 and MH058693.

Genotyping services were provided by the Center for Inherited Disease Research (CIDR). CIDR is fully funded through a federal contract from the National Institutes of Health to the Johns Hopkins University, contract N01-HG-65403.

Data and biomaterials were collected and supported by NIMH grant R01 MH59602 (to Miron Baron, M.D.) and by funds from the Columbia Genome Center and the New York State Office of Mental Health. The main contributors to this work were Miron Baron, M.D. (principal investigator), Jean Endicott, Ph.D. (co-principal investigator), Jo Ellen Loth, M.S.W., John Nee, Ph.D., Richard Blumenthal, Ph.D., Lawrence Sharpe, M.D., Barbara Lilliston, M.S.W., Melissa Smith, M.A., and Kristine Trautman, M.S.W., all from Columbia University Department of Psychiatry, New York. A small subset of the sample was collected in Israel in collaboration with Bernard Lerer, M.D., and Kyra Kanyas, M.S., from the Hadassah -Hebrew University Medical Center, Jerusalem. We are grateful to the patients and their family members for their cooperation and support and to the treatment facilities and other organizations that collaborated with us in identifying families.

The study was approved by the Wako First Research Ethics Committee of RIKEN.

Sequencing

All 4 exons of the *SLC25A4* gene were examined by PCR-direct sequencing. Primer sequences were as follows: Exon1F, AGCCCGGCGGGGATATAAG; Exon1R, GTGGCCTGGCGCAGATTTTC; Exon2F, TACCCTGCCTGTCCTCTG; Exon2R, TGTGAGTTATAGATCCCATA; Exon3F, GCAAGGTCAGAGCATGGA; Exon3R, TGTAAAATGTTCTCCTCGG; Exon4F, ATGCCCAGATGACCCTGATT; Exon4R, AACCACACAATGGATCTGTG. PCR were performed using LA Taq (Takara Bio, Shiga, Japan) with GC buffer I and GC buffer II. After each exon was amplified by PCR using Ex Taq (Takara Bio), the product was treated with ExoSAP-IT (Thermo Fischer Scientific, Waltham, MA, USA) and sequenced using a BigDye terminator kit (Thermo Fischer Scientific) with a primer used for

PCR amplification. The detected mutations were verified by sequencing the reverse strand. PCR was performed at 95ºC 3min, 95ºC 30 sec, 55ºC or 53ºC 30 sec, and 72ºC 30 sec for 38 cycles, followed by final extension at 72ºC 5 min. After PCR, the samples were stored at 15ºC. PCR was performed by ABI GeneAmp PCR System 9700.

Animals

All animal care and experimental procedure were in accordance with the guidelines for proper conduct of animal experiments published by Science Council of Japan and approved by RIKEN Wako Animal Experiment Committee and Genetic Recombinant Experiment Safety Committee. Floxed exon 2-3 of *Slc25a4* mouse line (*Slc25a4*$^{tm1a(EUCOMM)Wtsi}$) was obtained from International Knockout Mice Consortium (IKMC). *Slc25a4*$^{tm1a(EUCOMM)Wtsi}$ mice had been generated under the genetic background of C57BL/6NTac. The *Slc25a4*$^{tm1a(EUCOMM)Wtsi}$ mice were backcrossed with C57/BL6J mice for three generations. Flp-transgenic mouse line (B6 $^{Tg(cat-Flpe)36}$) was previously generated under the background of C57BL/6J [1]. Nestin-Cre transgenic mouse line (B6.Cg$^{Tg(Nes-cre)1Kln/J}$) was obtained from Jackson laboratory. B6.Cg$^{Tg(Nes-cre)1Kln/J}$ had been generated under the background of (C57BL/6 x SJL)F2, and after the introduction from Jackson Laboratory to RIKEN Brain Science Institute, it was backcrossed with C57BL/6J for at least five generations. These mice were housed on a 12-h light/dark cycle setting (light-off at 8:00 p.m./light-on at 8:00 a.m.) and with *ad libitum* access to regular chow (Oriental Yeast Co. Ltd., Tokyo, Japan) and water except for prior to or during several behavioral tests and were maintained by crossing with C57BL/6J mice (CLEA Japan Inc., Tokyo, Japan) under specific pathogen-free condition. Heterozygous for *Slc25a4*$^{tm1a(EUCOMM)Wtsi}$ mice were crossed with Flp-transgenic mouse line to generate the mice whose LacZ and Neomycin resistance genes flanked by FRT sequence was removed but exon 2-3 of *Slc25a4* flanked by loxP sequence was intact (*Slc25a4*$^{fl/+}$). The mice heterozygous for floxed exon 2-3 of *Slc25a4* (*Slc25a4*$^{fl/+}$) were crossbred with Nestin-Cre transgenic mice to produce *Slc25a4*$^{fl/+}$; Nes-Cre^{+} mice. To generate the mice homozygous and heterozygous cKO for *Slc25a4* with Nestin-Cre allele (*Slc25a4*$^{fl/fl}$; Nes-Cre^{+} and *Slc25a4*$^{fl/+}$; Nes-Cre^{+}), female *Slc25a4*$^{fl/fl}$ or *Slc25a4*$^{fl/+}$ mice and male *Slc25a4*$^{fl/+}$; Nes-Cre^{+} mice were mated. The number of mice used for behavioral screening by the IntelliCage was decided based on the recommendation for this apparatus. The number of the mice used for 5-CSRTT was determined by power calculation. To examine the direct effect of gene knockout, at least 3 mice in each group was used for the analysis. Most of the experiments were performed unblinded.

Cloning of 3xFLAG tagged *Ant1* mutant form

The EGFP sequence in pEGFP N1 vector (Takara Bio) was substituted to multiple cloning site

with 3xFLAG sequence at EcoRI and NotI site. Human *SLC25A4* cDNA was amplified from cDNA library of SH-SY5Y cell line derived from human neuroblastoma by PCR with HindIII site and Kozak sequence-linked forward primer and BamHI site-linked reverse primer which contained the sequence before stop codon of wild type and nonsense mutation form of *SLC25A4*. The PCR product was ligated with the HindIII and BamHI site of p3xFLAG vector. Site directed mutagenesis was performed by using PrimeSTAR polymerase (Takara Bio) with the primer listed in the section of primers. The sequence of the cloning vectors was validated by the Sanger sequencing using DNA analyzer (ABI 3730xl, Thermo Fisher scientific). Primers are shown in the section of the primers.

Cell culture and transfection

Neuro 2a cells were obtained from Japanese Collection of Research Bioresources (JCRB) Cell Bank and cultivated in DMEM (Sigma-Aldrich) containing 10% FBS in 5% CO_2 incubator at 37˚C. Transfection was conducted by using Lipofectamine 2000 (Thermo Fisher scientific) for Neuro 2a cells.

Immunoblotting

Proteins in cultivated cells were lysed with RIPA buffer which was consisted of 150 mM NaCl (Wako Pure Chemical Industries, Osaka, Japan), 1% NP-40 (Sigma-Aldrich), 1% doxycholate (Sigma-Aldrich), 0.1% SDS (Wako Pure Chemical Industries), 1x protease inhibitor cocktails (Sigma-Aldrich) in 25 mM Tris buffer, pH 7.5 (Sigma-Aldrich). Protein lysates were mixed with the same volume of 2 x Laemmli buffer which was consisted of 4% SDS, 10% 2-mercaptoethanol, 20% glycerol, bromophenol blue in 125 mM Tris buffer (pH 6.8) and were loaded onto acrylamide gel (Wako Pure Chemical Industries). After size separation by electrophoresis, proteins were transferred to PVDF membranes (Merck Millipore, Darmstadt, Germany). The membranes were blocked with 50 mM Tris buffer containing 150 mM NaCl, 0.05% Tween-20 and 5% skim milk. Primary antibodies including anti-FLAG (Medical and Biological Laboratories, Aichi, Japan, FLA-1, 1:1000) and anti-Tim23 (BD Biosciences, Franklin Lakes, NJ, USA, 32/Tim23, 1:5000) and secondary antibodies including HRP conjugated anti-Mouse IgG (GE Healthcare, Little Chalfont, UK, 1:2000) diluted in blocking buffer were used. After adding ECL reagent (GE Healthcare), images were captured by LAS3000 (Fuji film Corp., Tokyo, Japan)

Immunohistochemistry

For immunohistochemistry, mouse brains were fixed by cardiac perfusion with saline followed by 4% PFA and post fixation in 4% PFA at 4˚C overnight. The fixed brains were immersed in 30% sucrose in 0.1 M PBS for two days and embedded in OCT compound (Sakura Finetech Japan,

Tokyo, Japan). Cryosections of the brains were prepared by cryostats (Leica CM 3050S, Leica Biosystems, Wetzlar, Germany) and were mounted on slide glass (Matsunami Glass, Osaka, Japan). Antigen retrieval was conducted by autoclave with 10 mM Citrate buffer at 80˚C for 20 min. The slides were treated with 0.1 M PBS containing 0.1% Triton X-100 and 5% normal donkey serum (blocking solution) for 30 min for the purpose of permeabilization and blocking. Primary antibodies including anti-MTCO1(Thermo Fisher scientific, 1D6E1A8, 1:500), SDHA (Thermo Fisher scientific, 2E3GC12FB2AE2, 1:2000) and TPH2 (Sigma-Aldrich, HPA046274, 1:500) and secondary antibodies including Alexa Fluor 488 conjugated goat anti-mouse IgG1 (Thermo Fisher scientific, A-21121, 1:500), Alexa Fluor 546 conjugated goat anti-mouse IgG2a (Thermo Fisher scientific, A-21133, 1:500) and DyLight 648 conjugated donkey anti-rabbit IgG (Jackson Immuno research laboratory, 1:500) diluted in blocking solution were used for detection. Cryosections were covered by cover slip with PermaFluor aqueous mounting medium (Thermo Fisher scientific). Images for IHC were captured by confocal microscopy IX81 with FV1000 (Olympus Corporation, Tokyo, Japan), Observer Z1 with AxioVision 4.6 (Zeiss, Oberkochen, Germany) or Nano Zoomer Digital Pathology (NDP) system (Nanozoomer 2.0RS and S360, Hamamatsu Photonics, Hamamatsu, Japan).

Fluorescent *in situ* hybridization (FISH)

FISH was conducted as previously described [2]. DIG labeled antisense RNA probe was generated by using DIG RNA labeling kit (Sigma-Aldrich) with the primers listed in the section of primers. Fast red (Thermo Fisher scientific) was used for the detection of *Slc25a4* mRNA. Images were captured by NDP.

Mouse behavioral screening with IntelliCage

The IntelliCage apparatuses (NewBehavior AG, Zurich, Switzerland) were used for behavioral screening as described previously [3, 4]. Male mice including 8 heterozygous cKO mice (*Slc25a4*$^{fl/+}$; Nes-Cre^{+}), 10 homozygous cKO mice (*Slc25a4*$^{fl/fl}$; Nes-Cre^{+}) and 8 controls (*Slc25a4*$^{fl/+}$ or *Slc25a4*$^{fl/fl}$; Nes-Cre^{-}), which were 20-27 week old, were used. Radiofrequency transponders (Trovan Ltd., http://www.trovan.com/) were subcutaneously transplanted into the neck of the mice under isoflurane (Wako Pure Chemical Industries) inhalational anesthesia. After one week recovery period, group housing of 6-8 mice which were grouped by impartial genotypes in an IntelliCage was started. On the first day, the mice were freely able to access to the drinking water bottles that were set as couples in each four corners, without closing front doors. For the following three days, the mice were trained to open the doors in front of water nozzles for five sec by nosepoking into the caves in front of the doors. In the following several trials, conditions to open the doors by nosepoking were spatially and temporally limited to evaluate the traits of the mice.

Since the transponders on two control mice did not accurately work to collect count of visits to the drinking corners and nose pokes in the caves even though they precisely lead to open the doors by nosepoking due to unknown defect, the data obtained from these two mice were excluded.

-Reward based place preference learning and reversal learning

In advance of this trial, the mice were trained to learn that the time of water supply was restricted to three-hour drinking session from 9:00 p.m. (one hour after lights off) per day for three days. In place preference learning trial, in addition to the time restriction, the corner where each mouse was able to drink water was also restricted to one among all four corners in an IntelliCage for seven days. In the next reversal learning, the setting of the restricted corner was switched to the opposite corner and the ability to flexible response of the mice were measured for five days. The learning of the place was evaluated by the correct visit rates which were quotients of correct corner visits/all corner visits during drinking session.

- Impulsivity and attention test

In advance of this trial, the mice were trained to learn that yellow light signal indicated above the doors was the sign of the period when the sensor to open the door was active over a week. In the first three days, lighting was randomly started at 1, 2 or 4 sec after corner entry and was held for four sec. In the next five days, the timing of the lighting was fixed at two sec after a mouse visit, if a mouse, however, conducted nose poke prior to the light-ON (premature nose poke), lighting would be canceled as fail of the trial (punishment). And the next five days, the timing of the lighting was fixed at four seconds after corner entry but the limited hold was randomly selected from 0.5, 1 and 2. In this test, the timing of the lighting was fixed at four second and the limited hold was randomly selected from 0.3, 0.5, 1 and 2. Also during the test, the lighting would be canceled by premature nose poke. The drinking session was set from 9:00 p.m. to 8:00 a.m. during this training and test. The actions of the mice had been recorded for two days and the attention to short lighting was evaluated by the success rates which were quotients of correct nose pokes/total visits.

-Delay discounting task

In this task, the two bottles containing 0.5% sodium saccharin (Merck Millipore) water or purified water were juxtaposed in each corner. In advance of this trial, the mice were allowed to freely access all bottles by nosepoking to remember the side of 0.5% sodium saccharin water for four days. The last day of this habituation period was assigned to the day in which delay until the activation of the sensor to open the windows in saccharin side was 0. After the habituation period, the delay until the activation of the sensor to open the windows in saccharin side was elongated as the order of 0.1 and 1 to 8 seconds with increments of 1 second day by day. Discounting of saccharin reward by delay was evaluated by the saccharin nose poke rates which were quotients of nose poke to saccharin side in total nose poke and licking duration of each bottle.

Saccharin preference test

After behavioral analysis by using IntelliCage, these mice were applied to conventional saccharin preference test. Mice were deprived of water from 18:00 on the day before the test. On the day of the test, two bottles containing 0.5 % sodium saccharin (Merck Millipore) water or purified water were placed on the home cage for 30 minutes. Heterozygous cKO mice (*Slc25a4*$^{fl/+}$; Nes-Cre^{+}) (n = 7), homozygous cKO mice (*Slc25a4*$^{fl/fl}$; Nes-Cre^{+}) (n = 10), and control mice (*Slc25a4*$^{fl/+}$ or *Slc25a4*$^{fl/fl}$; Nes-Cre^{-}) (n = 8) were used.

5-choice serial reaction time task (5-CSRTT)

The 5-CSRTT operant chamber (O'HARA & Co., Tokyo, Japan) was used as previously described with minor modification [5]. Male mice including 8 controls (*Slc25a4*$^{fl/+}$ or *Slc25a4*$^{fl/fl}$; Nes-Cre^{-}), 8 heterozygous cKO mice (*Slc25a4*$^{fl/+}$; Nes-Cre^{+}) and 8 homozygous cKO mice (*Slc25a4*$^{fl/fl}$; Nes-Cre^{+}), which were 8-13 week old at the beginning of training, were given restricted food to reduce their body weight by approximately 85% of free-feeding body weight. At first, the mice were trained to acquire 30 test diet pellets (SLC Japan, Hamamatsu, Japan) which were delivered on a fixed interval 20 or 30 seconds from pellet tray in consecutive two days. Training of the mice to acquire sugar pellets by precise nosepoking followed the parameter setting shown below until the mice met the criterion of each step. After the accomplishment of TR-7, the mice were examined in the test as the following order to minimize the effect of parameter alteration in the test: standard, long inter-trail interval (ITI), long stimulation duration (SD), short SD and short ITI. The mice used for this behavioral test were also used for the analysis of gene expression analysis of *Maob*, *Tph2*, and *Slc6a4* in DR by quantitative real time PCR and at 34 - 39 week old. The parameters of the 5-CSRTT test are summarized below.

Schedule, Session Time (s) , No. of Trials, ITI (s), Range (s), Increment (s), Perseverative (s), Premature (s), Limited Hold (s), Stimulus Duration (s), Punishment Delay (s), Criterion.

TR-1: 1800, 100, 2, 0, 0, 1, 1, 30, 30, 2, >70.

TR-2: 1800, 100, 2, 0, 0, 1, 1, 15, 15, 2, >70.

TR-3: 1800, 100, 3, 0, 0, 1, 1, 15, 15, 3, >70.

TR-4: 1800, 100, 3, 0, 0, 1, 1, 5, 2, 3, >70.

TR-5: 1800, 100, 3, 0, 0, 1, 1, 5, 2, 3, >70.

TR-6: 1800, 100, 5, 0, 0, 1, 3, 5, 2, 5, >70.

TR-7: 1800, 100, 5, 0, 0, 1, 3, 5, 1, 5, >50.

Long ITI: 1800, 100, 8, 0, 0, 4, 4, 5, 1, 5.

Long SD: 1800, 100, 5, 0, 0, 2, 3, 5, 2.5, 5.

Short SD: 1800, 100, 5, 0, 0, 2, 3, 5, 0.5, 5.

Short ITI: 1800, 100, 2, 0, 0, 1, 1, 5, 1, 5.

Short variable ITI: 1800, 100, 3, 2, 1, 1, 2, 5, 1, 5.

Long variable ITI: 1800, 100, 8, 2, 1, 4, 4, 5, 1, 5.

Quantification of mtDNA deletion and mtDNA copy number

DNA was extracted from the dorsal raphe using QIAamp DNA micro kit (Qiagen, Hilden, Germany). Partially deleted mitochondrial DNA (ΔmtDNA) was measured by quantitative PCR methods using SYBR Premix Ex Taq Kit (Takara Bio). ΔmtDNAs were amplified by a primer pair (D3, 5´-CGA TAT ACA TAA ATG TAC TGT TGT ACT ATG-3´ and D4, 5´-AAC TCT AAT CAT ACT CTA TTA CGC-3´) with short extension time as described [6]. The *Nd4* region amplified by a primer pair 5´-CAT CAC TCC TAT TCT GCC TAG CAA-3´ and 5´-AGT CCT CGG GCC ATG ATT ATA GT-3´ was used as a reference. Copy number of mtDNA was measured by *Nd4* region with a reference of nuclear gene, *RNaseP* (5′ -CTC CCC AAA TGG AAG ATG AG-3′ and 5′ -TAT TCT ACG TTC CGG TGT GG-3′). After an initial denaturation step (95 °C for 30 s), PCR amplification for 40 cycles (95 °C for 5 s; 60 °C for 30 s) was performed using QuantStudio 12K Flex (Thermo Fisher Scientific). For quantification of mtDNA deletion 30 - 39 week old male mice were used. For mtDNA copy number analysis, 54 -56 week old male mice were used. Control mice were *Slc25a4*$^{fl/+}$ or *Slc25a4*$^{fl/fl}$ without Nestin-Cre.

Quantification of *Ant1/Ant2* mRNAs

mRNA expression of *Ant1* (*Slc25a4*) and *Ant2* (*Slc25a5*) were measured by quantitative PCR methods using SYBR Premix Ex Taq Kit (Takara Bio). After an initial denaturation step (95 °C for 30 s), PCR amplification for 40 cycles (95 °C for 5 s; 60 °C for 30 s) was performed using QuantStudio 12K Flex (Thermo Fisher Scientific). For these analysis, 78 - 114 week old female mice were used (n = 3 for each group).

Isolation of mitochondria

Brain mitochondria were isolated using a discontinuous Percoll gradient developed by Sims [7] with minor modifications [8]. Briefly, a male mouse of each genotype was decapitated and the brain was transferred to an ice-cold isolation buffer (320 mM sucrose, 1 mM EGTA, and 10 mM MOPS; pH 7.4). The forebrain tissue was dissected and homogenized in 12% Percoll with a Dounce homogenizer. The homogenate was layered on a discontinuous gradient of 26% and 40% Percoll (GE Healthcare) in the isolation buffer and centrifuged in at 30,700 g for 5 min at 4 °C, and washed two times in the isolation buffer at 16,700 g for 5 min and 7,300 g for 10 min, respectively. Fatty acid-free bovine serum albumin (Sigma-Aldrich) was added at the last washing step (final 0.1%). The mitochondrial pellet was suspended in a buffer (210 mM sucrose, 20 mM KCl, 3 mM glycylglycine, and 1 mM KH_2PO_4 (pH 7.2)) treated with Chelex 100 resin (Bio-Rad, Richmond,

CA). Mitochondrial protein concentration was determined by the Bradford protein assay in the microplate protocol (Nacalai tesque, Kyoto, Japan) and adjusted to 150 µg/ml in a Ca^{2+}-free buffer. Heterozygous cKO mice (*Slc25a4*$^{fl/+}$; Nes-Cre^{+}) (n = 4), homozygous cKO mice (*Slc25a4*$^{fl/fl}$; Nes-Cre^{+}) (n = 4), and control mice (*Slc25a4*$^{fl/fl}$; Nes-Cre^{-}) (n = 3) aged 8 to 27 weeks were used this analysis.

Measurement of calcium retention capacity

Extra-mitochondrial free Ca^{2+} concentration ($[Ca^{2+}]_{exm}$) was monitored with 200 nM Calcium Green-5N (Thermo Fisher Scientific, Waltham, MA) (Ex 480 nm, Em 540 nm) in a 96 well plate at 30 °C in a Drug Screening System (FDSS 3000, Hamamatsu Photonics). The mitochondrial pellet was divided into 45 µl aliquots/ well and added 45 µl of Ca^{2+}-free buffer containing Calcium Green-5N, 20 mM glutamate plus 2 mM malate for analysis of Calcium retention capacity (CRC) (triplicate measurements). The total solution in each well was 90 µl in volume. The time resolution of the $[Ca^{2+}]_{exm}$ measurement was set at 0.48 s. To evaluate the CRC, 10 µl of Ca^{2+} solution was repeatedly added at 1-min intervals after preincubation time for 5 min and a baseline reading for 3 min. The stock solutions of 117 µM $CaCl_2$ gave final concentrations of 11.7 µM for one injection, which approximately corresponded to 173.3 nmol Ca^{2+}/mg mitochondrial protein. One genotype was assessed per day in a blind manner.

Quantification of monoamine in tissue by HPLC

The mice were euthanized by cervical dislocation and the brains were removed and placed on an ice-cold brain slicer matrix with 1 mm coronal section slice intervals (EM Japan, Tokyo, Japan). Tissues of the nucleus accumbens were dissected by using tweezers and microscissors (World Precision Instruments Inc., Sarasota, FL) under a wide-field binocular microscope (Olympus Corporation) and were immediately frozen in liquid nitrogen. Neurochemical components were extracted by sonication in 100 µl of 0.1 M perchloric acid (Wako Pure Chemical Industries) with 0.1 mM EDTA-2Na (Dojindo, Kumamoto, Japan). After removing of insoluble matter by centrifuge at 20,000 g for 15 min at 0°C and filtration through 0.45 µm PVDF membrane (Nacalai tesque), the samples were subjected to HPLC with a EICOMPAK SC-5ODS for separation of dopamine, noradrenaline and serotonin and their metabolites and electrochemical detector ECD-300 (Eicom Corporation, Kyoto, Japan). The solvent for mobile phase was composed of 13% methanol and 83% of 0.1M sodium acetate/citrate buffer (Wako Pure Chemical Industries, pH3.5) including 190 mg/L of 1-octanesulfonic acid (Nacalai tesque) and 5 mg/L of EDTA-2Na. For the measurement of monoamine levels, mice were given 0.5% saccharin water for the sake of conditioning of reward expectation. In this experiment, control mice included *Slc25a4*$^{+/+}$;Nestin-Cre^{+} and *Slc25a4*$^{+/+}$, *Slc25a4*$^{fl/+}$ or *Slc25a4*$^{fl/fl}$ without Nestin-Cre. For this experiment, 88 - 103 week old male mice

were used.

Electrophysiological analysis

Brain slices for experiments were prepared from 8-12-week-old, male mice as described previously [9]. The mice were deeply anesthetized with halothane inhalation (~2% in air, v/v). Following decapitation, the brains were rapidly removed and placed in ice-cold sucrose-based cutting solution (~4°C) that contained the following compounds; 252 mM sucrose, 21 mM $NaHCO_3$, 3.35 mM KCl, 0.5 mM $CaCl_2$, 6.0 mM $MgCl_2$, 0.6 mM NaH_2PO_4, and 10 mM glucose. The brain was blocked and three coronal slices (250 µM) were made by vibratome (VT1200S; Leica, Microsystems, Wetzlar, Germany) through the entire rostro-caudal extent of the DR, and placed in a submerged chamber for at least 1h in artificial cerebrospinal fluid (ACSF) that contained 138.6 mM NaCl, 3.35 mM KCl, 2 mM $CaCl_2$, 1.3 mM $MgCl_2$, 21.0 mM $NaHCO_3$, 0.6 mM $NaH_2Po_42H_2O$, and 10.0 mM glucose. ACSF was maintained at pH 7.4 by bubbling 95% O_2-5% CO_2 gas.

Individual slices were transferred to a recording chamber attached to a microscope stage and continuously perfused with oxygenated ACSF at a flow rate of 1.4 ml/min, maintained at 29°C. Borosilicate glass-patch electrodes (World Precision Instruments, Sarasota, FL, USA) were used for whole cell recordings from DR cells with resistance of 4-6 MΩ when filled with an internal solution of 150 mM potassium methanesulfonate, 1.0 mM KCl, 0.2 mM K-EGTA, 20 mM HEPES, 3.0 mM $MgATP_2$, 0.4mM Na-GTP and 15 mM biocytin (pH 7.38, ~295 mOsm). DR serotonergic cells were visually identified under IR-DIC images by using a water-immersion objective (40×, NA=0.80; Olympus, Tokyo, Japan).

Whole cell patch-clamp recordings were acquired and controlled using the Axon 700B Multiclamp amplifer (Molecular Devices, CA, US) and pClamp11 acquisition software (Molecular Devices, CA, US). The cell characteristics including resting membrane potential, input resistance, properties of action potential (AP) and input-output relationship were measured in the current-clamp mode. All signals were filtered at 2 kHz and sampled at 5–20 kHz. Obtained data were analyzed by using software, clampfit (Molecular Devices, CA, US) and Kyplot (Kyence, Tokyo, Japan). For this analysis, heterozygous *Ant1* cKO mice (*Slc25a4*$^{fl/+}$; Nes-Cre^{+}) (n = 4) and control mice (*Slc25a4*$^{fl/+}$; Nes-Cre^{-}) (n = 3) were used.

Primers used for cloning, site directed mutagenesis and real-time PCR

Primer sequences used for these experiments are as follows.

Primers for cloning

HindIII-Kozak-*hSLC25A4*-F, GGAAGCTTCAGCATGGGTGATCACGCTTGG

BamHI-*hSLC25A4* (-STOP)-R, ATGGATCCGACATATTTTTTGATCTCATC
BamHI-Q85X(-STOP)-R, ATGGATCCGGTGGGGAAGTAACGGATCACG
BamHI-Q175fs(-STOP)-R, ATGGATCCCAAAAATGTGCACGTTCTTGG

Primers for site directed mutagenesis
Q85X-F: CCCCACCTAAGCTCTCAACTTCGCCTTC,
Q85X-R: AGAGCTTAGGTGGGGAAGTAACGGATCA
A90D-F: AACTTCGACTTCAAGGACAAGTACAAGC,
A90D-R: CTTGAAGTCGAAGTTGAGAGCTTGGGTG
L98P-F: AAGCAGCCCTTCTTAGGGGGTGTGGATC,
L98P-R: TAAGAAGGGCTGCTTGTACTTGTCCTTG
D104G-F: GGTGTGGGTCGGCATAAGCAGTTCTGGC,
D104G-R: ATGCCGACCCACACCCCCTAAGAAGAGC
A114P-F: CTACTTTCCTGGTAACCTGGCGTCCGGT,
A114P-R: TTACCAGGAAAGTAGCGCCAGAACTGCT
Q175fs-F: GGCTCTACAGGGTTTCAACGTCTCTGTCC,
Q175fs-R: GAAACCCTGTAGAGCCCCCTCAGGCCATC
G246A-F: CGGAAAGCGGCCGATATTATGTACACGG,
G246A-R: ATCGGCCGCTTTCCGGCCGGACTGCATC
V289M-F: TGTATTGATGTTGTATGATGAGATCAAA,
V289M-R: TACAACATCAATACAAAAGCACCGCCCA

Primers for preparing DIG-labeled RNA probe for ISH of *Slc25a4*
T7-*Slc25a4*-f, TTGTAATACGACTCACTATAGGGGGATCAG
SP6-*Slc25a4*-r, GCTATTTAGGTGACACTATAGAATCAGCTC

Primers for real-time PCR

Ant1, F: TCCCTCTGCTTCGTCTACCC, R: CTCGCTGGGAAGATCCCTTG
Ant2, F: TCCTTGTGCTTTGTGTACCC, R: CCCTTTCAGCTCCAGCTTTG
Actb, F: CAGCAAGCAGGAGTACGATGAGTC, R: CAGTAACAGTCCGCCTAGAAGCAC
Maob, F: GGGCCAACCCAGAATCGTATC, R: GTATCAGCCGCTCAACTTCATT
Tph2, F: GTGACCCTGAATCCGCCTG, R: GGTGCCGTACATGAGGACT
Slc6a4, F: TATCCAATGGGTACTCCGCAG, R: CCGTTCCCCTTGGTGAATCT

Statistical analysis

Data were analyzed in Prism 4 (Graphpad software Inc., San Diego, CA) or IBM SPSS Statistics 20 (IBM Japan, Tokyo, Japan), or "R" (https://www.r-project.org/) or Kyplot (Kyence, Tokyo, Japan). For genetic association analysis, Fisher's exact probability test was used. In the comparison between the heterozygous or homozygous cKO mice and control mice, Student *t*-test was used. For place learning task and delay discounting task in IntelliCage, repeated measures ANOVA with Bonferroni’s post hoc test was used with main effects of genotype and delay or day. For other experiments, Student *t*-test and one way ANOVA with multiple comparison by Tukey’s method was used. The analysis was performed by genotypes, and no randomization process was involved in this study.

Supplementary Figure 1. Two loss-of-function mutations found in patients with bipolar disorder.

A, B) Sequence electropherogram of two mutations (A: p.Q85X, B: p.Q175RfsX38) found in patients with bipolar disorder.

C) Western blotting of 3xFLAG tagged wild type and mutant ANT1 proteins expressed in Neuro 2a cells. TIM23 (internal controls for inner mitochondrial membrane) was also blotted as internal control. Protein expression is markedly decreased for Q85X and Q175RfsX38.

D) Two NIMH families carrying p.Q85X or p.Q175RfsX38 are shown. Arrows indicate probands of the families. Genotyped individuals are indicated by plus or minus symbols, which denote the presence or absence of the *ANT1* mutation, respectively.

Supplementary Figure 2. Place learning and attention tests in the IntelliCage and other indices of five-choice serial reaction time task (5-CSRTT).

A-E) IntelliCage behavioral analysis of control (black, n = 6), heterozygous cKO (red, n = 8) homozygous cKO (blue, n = 10) mice.

A) Schematic drawing of place learning test in IntelliCage. A correct (light yellow) corner where mice could drink water was assigned to each mouse to examine the place discrimination leaning from other incorrect (grey) corners.

B) Place leaning and its reversal leaning in which the correct corner was converted to the opposite corner was examined for first 7 days and last 5 days, respectively. The data represents mean ± SEM.

C) Schematic drawing of the examination for impulsivity and attention. Mice were allowed to drink water by correct nose poke during LED-ON (0.3, 0.5, 1 or 2 seconds) starting at 4 second after corner entry.

D, **E**) Percentages of the trials in which mice did correct (D) or premature (E) nose poke response in total corner visits. The data represents mean ± SEM.

F) No significant difference in saccharin preference between genotypes (0.5% saccharin) (Control, n = 8; heterozygous cKO, n = 7; homozygous cKO, n = 10).

G-I) 5-CSRTT behavioral analysis of control (black, n = 8), heterozygous cKO (red, n = 8) homozygous cKO (blue, n = 8) mice.

G) Omission trials of heterozygous mutant mice was significantly increased compared with that of control mice in standard and long ITI tests. The data represents mean ± SEM.

H) Number of feed in heterozygous mutant mice was significantly decreased compared with that in control mice in standard test.

I) Reaction time in correct nose poke in heterozygous *Ant1* cKO mice was significantly longer compared with that in control mice in standard test.

** $P<0.05$, * $P<0.05$ by Student *t*-test.

Supplementary Figure 3. Effects of the Cre-transgene on the excitability of DR serotonergic neurons.
A) Representative action potentials (APs) of the DR neurons in the absence (left) and in the presence of Nes-Cre transgene (right) that were generated by injecting currents of 20 pA (black), 60 pA (blue), 100 pA (orange), and 140 pA (green), respectively. **B**) Pooled data of input-output relationship curves of the DR serotonergic neurons in the absence (open circles, n = 14 neurons from 1 mouse) and in the presence of Cre transgene (filled circles, n = 13 neurons from 1 mouse). APs were generated by injecting current steps from 0 pA to 160 pA in increments of 20 pA for 500 ms. The frequency of AP generation were plotted.

References

1. Kanki H, Suzuki H, Itohara S. High-efficiency CAG-FLPe deleter mice in C57BL/6J background. *Exp Anim* 2006; **55**(2)**:** 137-141.

2. Shimojo H, Ohtsuka T, Kageyama R. Oscillations in notch signaling regulate maintenance of neural progenitors. *Neuron* 2008; **58**(1)**:** 52-64.

3. Kobayashi Y, Sano Y, Vannoni E, Goto H, Suzuki H, Oba A *et al.* Genetic dissection of medial habenula-interpeduncular nucleus pathway function in mice. *Front Behav Neurosci* 2013; **7:** 17.

4. Masuda A, Kobayashi Y, Kogo N, Saito T, Saido TC, Itohara S. Cognitive deficits in single App knock-in mouse models. *Neurobiol Learn Mem* 2016; **135:** 73-82.

5. Asinof SK, Paine TA. The 5-choice serial reaction time task: a task of attention and impulse control for rodents. *J Vis Exp* 2014; (90)**:** e51574.

6. Kasahara T, Takata A, Kato TM, Kubota-Sakashita M, Sawada T, Kakita A *et al.* Depression-like episodes in mice harboring mtDNA deletions in paraventricular thalamus. *Mol Psychiatry* 2016; **21**(1)**:** 39-48.

7. Sims NR. Rapid isolation of metabolically active mitochondria from rat brain and subregions using Percoll density gradient centrifugation. *J Neurochem* 1990; **55**(2)**:** 698-

707.

8. Kubota M, Kasahara T, Nakamura T, Ishiwata M, Miyauchi T, Kato T. Abnormal Ca^{2+} dynamics in transgenic mice with neuron-specific mitochondrial DNA defects. *J Neurosci* 2006; **26**(47)**:** 12314-12324.

9. Gocho Y, Sakai A, Yanagawa Y, Suzuki H, Saitow F. Electrophysiological and pharmacological properties of GABAergic cells in the dorsal raphe nucleus. *J Physiol Sci* 2013; **63**(2)**:** 147-154.

Supplementary Figure 1

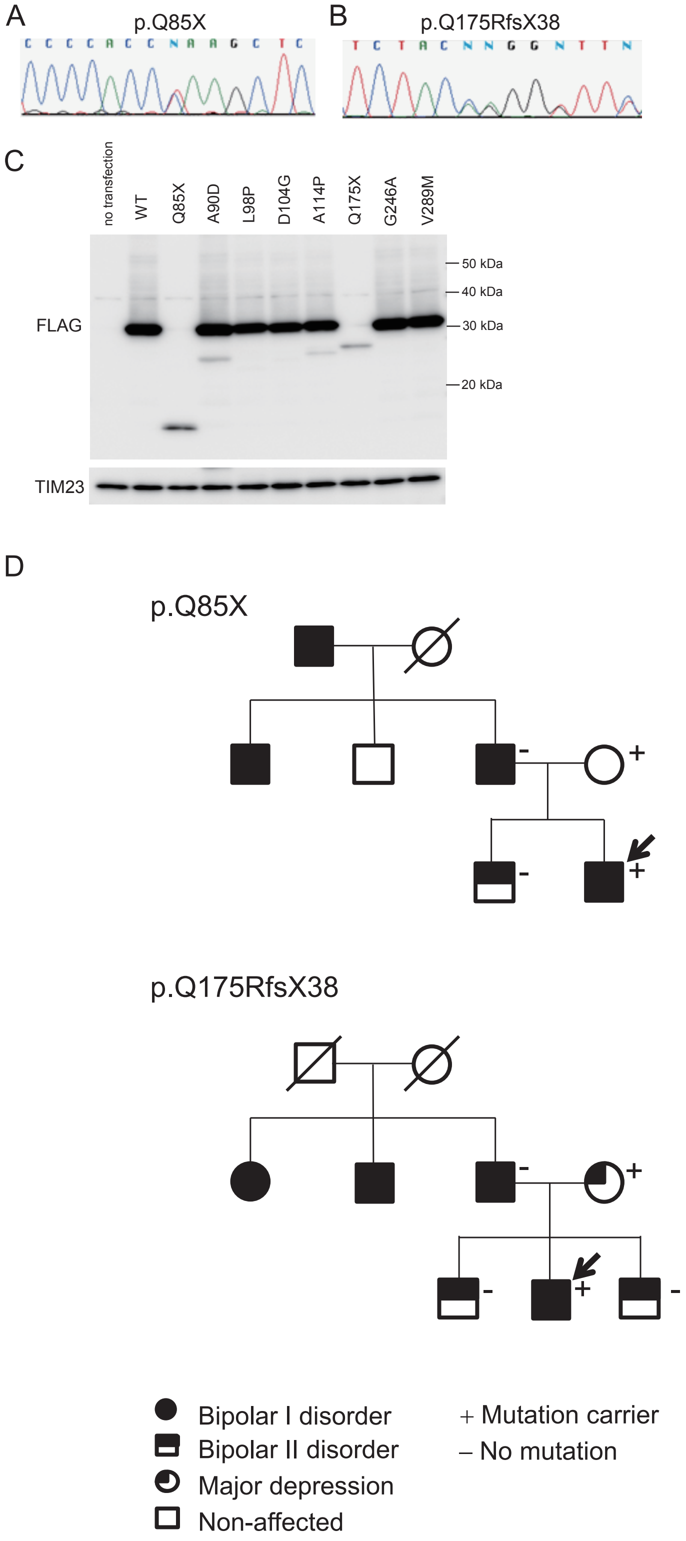

Supplementary Figure 2

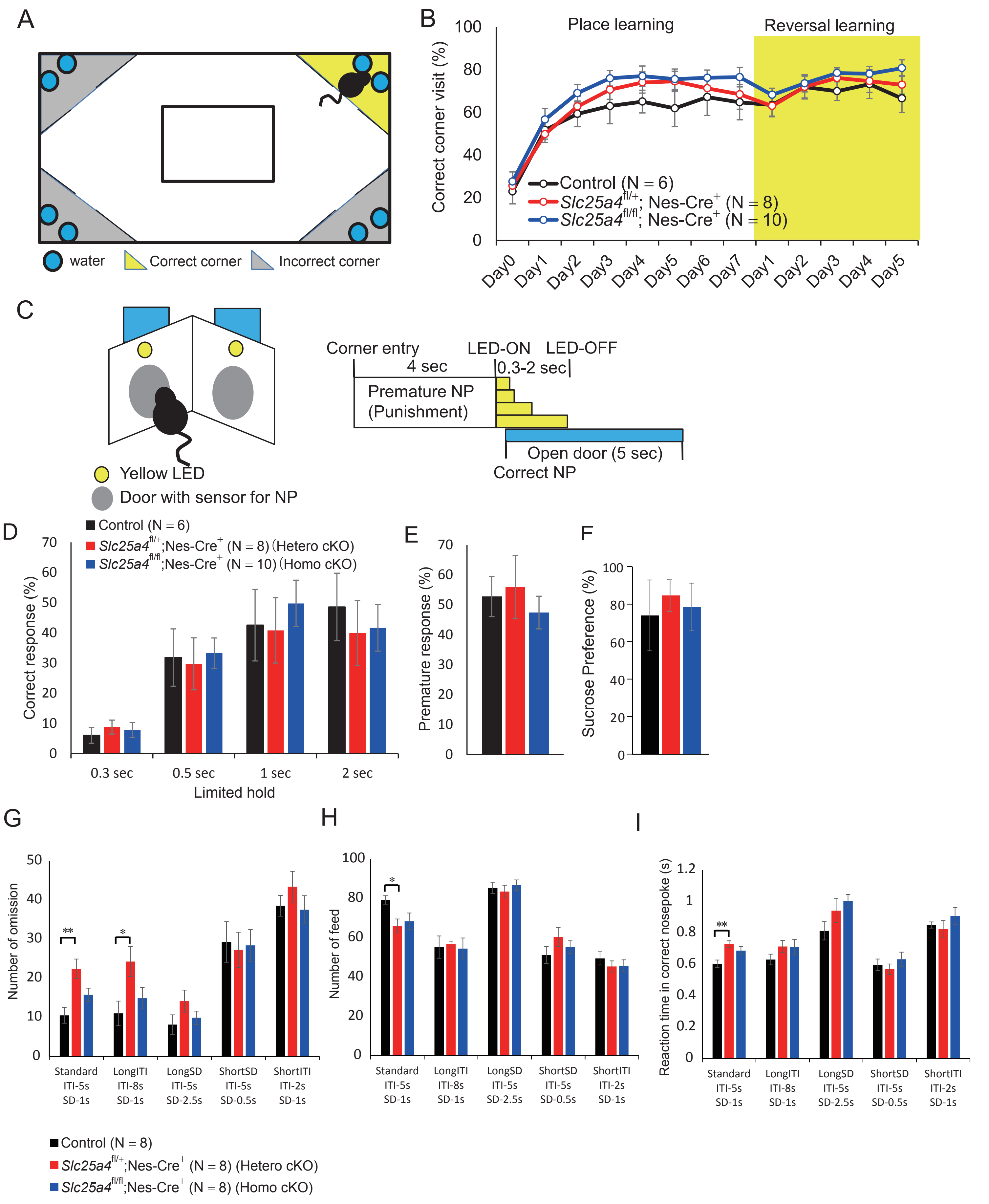

Supplementary Figure 3

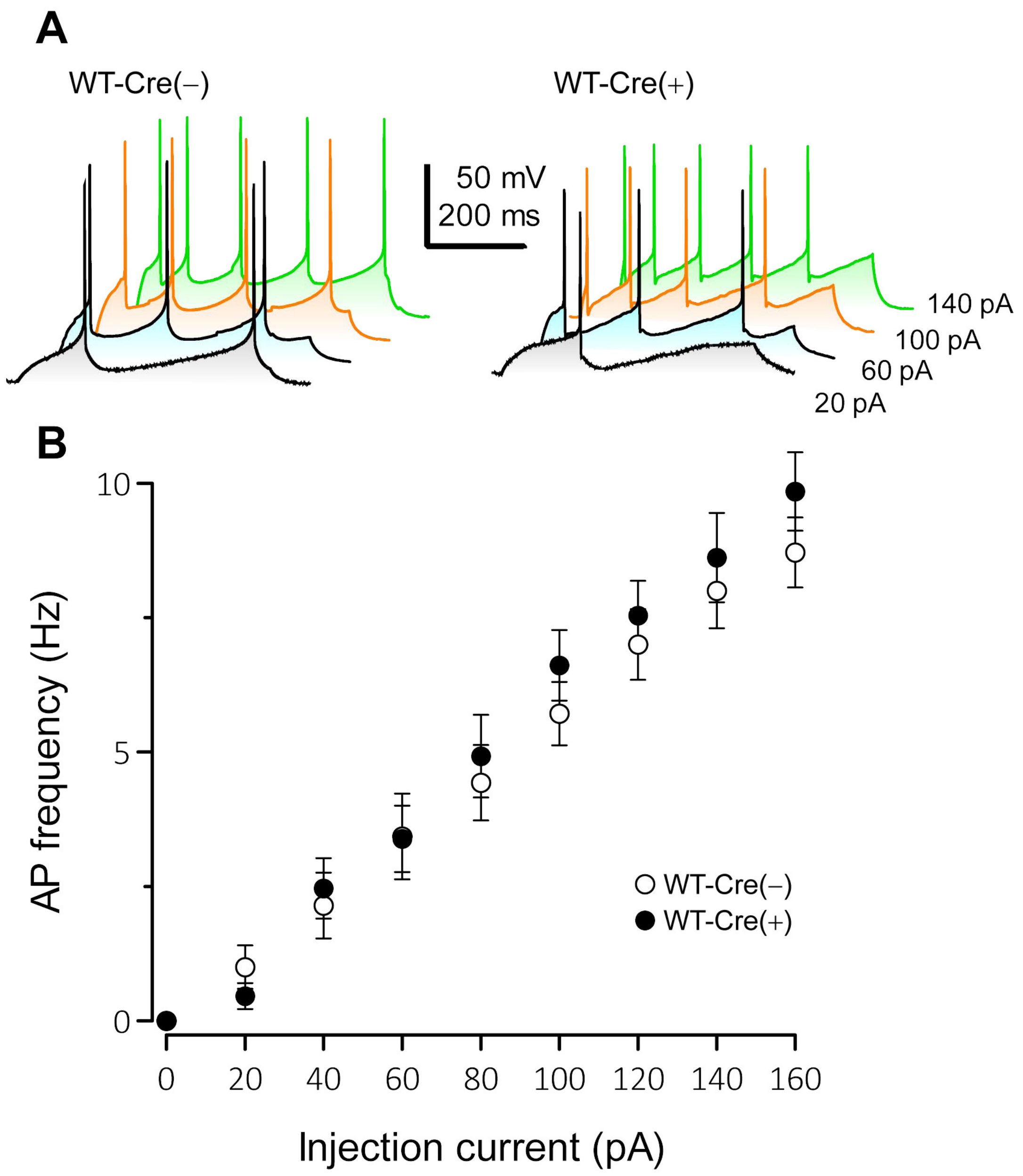

Supplementary Table 1. Electrophysiological properties of DR serotonergic neurons in heterozygous *Ant1* cKO mice.

	**Membrane properties**			**Action potential properties**				
	**Resting membrane potential (mV)**	**Input resistnace (MΩ)**	**Membrane capacitance (pF)**	**Overshoot (mV)**	**Threshold (mV)**	**fAHP (mV)**	**sAHP (mV)**	**Half-width (ms)**
Control	-73.5 ± 1.17 (44)	559.8 ± 19.8 (42)	68.2 ± 2.44 (14)	37.6 ± 0.78 (44)	-35.1 ± 0.50 (44)	4.37 ± 0.48 (44)	16.2 ± 0.61 (44)	1.59 ± 0.04 (44)
Hetero cKO	-71.8 ± 1.23 (48)	563.4 ± 25.7 (45)	65.1 ± 5.19 (16)	37.5 ± 0.65 (48)	-35.2 ± 0.43 (48)	3.98 ± 0.53 (48)	15.9 ± 0.64 (48)	1.60 ± 0.04 (48)
DF	90	85	28	90	90	90	90	90
t-value	1.03	0.11	0.52	0.11	0.12	0.55	0.33	-0.04
P-value	0.31	0.91	0.61	0.91	0.9	0.33	0.74	0.97

Values indicate mean ± standard error of the mean.
The numbers of data are indicated within parentheses.
DF: degrees of freedom; fAHP, fast afterhyperpolarization; sAHP, slow afterhyperpolarization.

Supplementary Table S2. Statistical results

Values to be tested	Values	Statistical tests	Statistical values
Supplementary Figure 1A-B	Number of subjects with LOF mutations of ANT1	Fisher's exact probability test	P=0.00040
Figure 1D	cortex, Ant1/Actb	one way ANOVA	df=2, F=28.6, p=0.001
Figure 1D	hippocampus, Ant1/Actb	one way ANOVA	df=2, F=16.0, p=0.004
Figure 1E	cortex, Ant2/Actb	one way ANOVA	df=2, F=0.896, p=0.457
Figure 1E	hippocampus, Ant2/Actb	one way ANOVA	df=2, F=1.946, p=0.223
Figure 1G	mitochondrial calcium retention capacity	One way ANOVA with multiple comparison by Bonferroni correction	df=2, F=4.356, p=0.023<br>Post hoc analysis:<br>Homo vs control: p=0.020,<br>Hetero vs control: p=0.476
Supplementary Figure 2B	Place Learning	Repeated measures ANOVA	Day: df=7, F=59,726, p=0.000,<br>Genotype: df=2, F=1.407, p=0.267,<br>Interaction: df=7, F=0.452, p=0.954
Supplementary Figure 2B	Reversal Learning	Repeated measures ANOVA	Day: df=4, F=11.880, p=0.000,<br>Genotype: df=2, F=0.684, p=0.515,<br>Interaction: df=8, F=1.430, p=0.196
Supplementary Figure 2D	Correct nose poke response in total corner visits	Repeated measures ANOVA	Time: df=3, F=18.813, p=0.000,<br>Genotype: df=2, F=0.094, p=0.910,<br>Interaction: df=6, F=0.279, p=0.944
Supplementary Figure 2E	Premature responses	one way ANOVA	df=2, F=0.343, p=0.714
Figure 2B	Saccharin nosepoke in total nosepoke (Delay discounting)	Repeated measures ANOVA	Delay: df=9, F=25.344, p=0.000,<br>Genotype: df=2, F=15.293, p=0.000,<br>Interaction: df=18, F=8.789, p=0.000.

Figure 2B (post hoc analysis)		One way ANOVA with multiple comparison by Bonferroni correction	Delay 0s: df=2, F=0.439, p=0.650,<br>Delay 0.1s: df=2, F=0.448, p=0.645,<br>Delay 1s: df=2, F=2.518, p=0.104,<br>Delay 2s: df=2, F=5.609, p=0.11,<br>Delay 3s: df=2, F=6.497, p=0.006,<br>Bonferroni: Hetero vs C, p=0.018, Homo vs C, p=0.008,<br>Delay 4s: df=2, F=9.493, p=0001,<br>Bonferroni: Hetero vs C, p=0.003, Homo vs C, p=0.002,<br>Delay 5s: df=2, F=10.071, p=0.001,<br>Bonferroni: Hetero vs C, p=0.002, Homo vs C, p=0.001<br>Delay 6s: df=2, F=11.977, p=0.000,<br>Bonferroni: Hetero vs C, p=0.001, Homo vs C, p=0.001<br>Delay 7s: df=2, F=13.158, p=0.000,<br>Bonferroni: Hetero vs C, p=0.000, Homo vs C, p=0.001<br>Delay 8s: df=2, F=21.029, p=0.000,<br>Bonferroni: Hetero vs C, p=0.000, Homo vs C, p=0.000
Supplementary Figure 2F	Sucrose preference	one way ANOVA	df=2, F=1.063, p=0.363
Figure 2D	Number of premature nosepoke	Student t-test, two-tailed (hetero vs C)	Standard: df=14, t=0.16, p=0.875,<br>LongITI: df=14, t=1.596, p=0.132,<br>LongSD: df=14, t=2.147, p=0.0497,<br>Short SD: df=14, t=1.200, p=0.250,<br>ShortITI: df=14, t=0.269, p=0.792
Figure 2E	Accuracy	Student t-test, two-tailed (hetero vs C)	Standard: df=14, t=1.543, p=0.145,<br>LongITI: df=14, t=3.469, p=0.00375,<br>LongSD: df=14, t=1.474, p=0.163,<br>Short SD: df=14, t=1.437, p=0.173,<br>ShortITI: df=14, t=0.067, p=0.948
Figure 2F	Number of perseverative responses	Student t-test, two-tailed (hetero vs C)	Standard: df=14, t=1.724, p=0.106,<br>LongITI: df=14, t=2.128, p=0.0515,<br>LongSD: df=14, t=1.000, p=0.334,<br>Short SD: df=14, t=0.521, p=0.610,<br>ShortITI: df=14, t=1.011, p=0.3292
Supplementary Figure 2G	Number of omission	Student t-test, two-tailed (hetero vs C)	Standard: df=14, t=3.671, p=0.00251,<br>LongITI: df=14, t=2.668, p=0.0183,<br>LongSD: df=14, t=1.590, p=0.134,<br>Short SD: df=14, t=0.291, p=0.775,<br>ShortITI: df=14, t=1.028, p=0.321

Supplementary Figure 2H	Number of Feed	Student t-test, two-tailed (hetero vs C)	Standard: df=14, t=3.188, p=0.00657,<br>LongITI: df=14, t=.0.255 p=0.802,<br>LongSD: df=14, t=0.432, p=0.672,<br>Short SD: df=14, t=1.403, p=0.182,<br>ShortITI: df=14, t=0.932, p=0.367
Supplementary Figure 2I	Reaction time in correct nosepoke	Student t-test, two-tailed (hetero vs C)	Standard: df=14, t=3.959 p=0.00142,<br>LongITI: df=14, t=1.624 p=0.126,<br>LongSD: df=14, t=1.343, p=0.200,<br>Short SD: df=14, t=0.574, p=0.575,<br>ShortITI: df=14, t=0.434, p=0671
Figure 3C	mtDNA deletions	Student t-test, two-tailed	Hetero vs C: df=14, t=1.543, p=0.145<br>Homo vs C: df=16, t=1.758, p=0.098
Figure 3D	Relative mtDNA copy number	Student t-test, two-tailed	Hetero vs C: df=4, t=4.759, p=0.00891,<br>Homo vs C: df=4, t=2.393, p=0.0749
Figure 3E	5-HIAA/5-HT	Student t-test, two-tailed	Hetero vs C: df=9, t=4.100, p=0.00267,<br>Homo vs C: df=9, t=1.196, p=0.262
Results, Serotonergic dysfunction in Ant1 KO mice	5-HIAA/5-HT	Student t-test, two-tailed	Cre(+) vs Cre (-): df=4, t=1.743,p=0.156
Figure 3F	Maob/Tph2	Student t-test, two-tailed	Hetero vs C: df=4, t=3.336, p=0.0156,<br>Homo vs C: df=4, t=1.153, p=0.286
Figure 3G	Maob/Slc6a4	Student t-test, two-tailed	Hetero vs C: df=4, t=3.645, p=0.0107,<br>Homo vs C: df=4, t=0.117, p=0.909
Figure 4B	Frequency of action potentials	Repeated measures ANOVA (Greenhouse-Gersser)	Current: df=1.701, F=529.950, p=0.000,<br>Genotype: df=1, F=3.241, p=0.075,<br>Interaction: df=1.701, F=3.163, p=0.053
		Student t-test, two-tailed (hetero vs control)	0pA: df=92, t=-0.888, p=0.377,<br>20pA: df=92, t=-1.407, p=0.163,<br>40pA: df=92, t=-1.102, p=0.273,<br>60pA: df=92, t=-1.384, p=0.170,<br>80pA: df=92, t=-1.704, p=0.092,<br>100pA: df=92, t=-1.867, p=0.065,<br>120pA: df=92, t=-1.79, p=0.077,<br>140pA: df=92, t=-1.985, p=0.050,<br>160pA: df=92, t=-2.021, p=0.046,
Supplementary Figure 3B	Frequency of action potentials	Repeated measures ANOVA (Greenhouse-Gersser)	Current: df=2.930, F=187.511, p=0.000,<br>Cre: df=1, F=0.265, p=0.611,<br>Interaction: df=2.930, F=1.120, p=0.346
